# A stable and sensitive Au metal organic frameworks resonance Rayleigh scattering nanoprobe for detection of SO_3_^2–^ in food based on fuchsin addition reaction

**DOI:** 10.3389/fnut.2022.1019429

**Published:** 2022-11-10

**Authors:** Xiaowen Lv, Yue Liu, Shuangshuang Zhou, Menglei Wu, Zhiliang Jiang, Guiqing Wen

**Affiliations:** ^1^Key Laboratory of Ecology of Rare and Endangered Species and Environmental Protection (Guangxi Normal University), Ministry of Education, Guilin, China; ^2^Guangxi Key Laboratory of Environmental Pollution Control Theory and Technology for Science and Education Combined with Science and Technology Innovation Base, Guilin, China

**Keywords:** sulfite, AuMOF nanoprobe, fuchsin addition reaction, resonance Rayleigh scattering, energy transfer

## Abstract

A stable Au metal organic frameworks (AuMOF) nanosol was prepared. It was characterized by electron microscopy and molecular spectral techniques. In pH 6.8 PBS buffer solution, AuMOF nanoprobes exhibit a strong resonance Rayleigh scattering (RRS) peak at 330 nm. After basic fuchsin (BF) adsorbing on the surface of AuMOF, the RRS energy of the nanoprobe donor can be transferred to BF receptor, resulting in a decrease in the RRS intensity at 330 nm. Both sulfite and BF taken place an addition reaction to form a colorless product (SBF) that exhibit weak RRS energy transfer (RRS-ET) between AuMOF and SBF, resulting in the enhancement of the RRS peak. As the concentration of SO_3_^2–^increases, the RRS peak is linearly enhanced. Thus, a new and sensitive RRS-ET method for the detection of SO_3_^2–^ (0.160–5.00 μmol/L) was developed accordingly using AuMOF as nanoprobes, with a detection limit of 0.0800 μmol/L. This new RRS method was applied to determination of SO_3_^2–^ in food and SO_2_ in air samples. The recoveries of food and air samples were 97.1–106% and 92.9–106%, and the relative standard deviation (RSD) was 2.10–4.80% and 2.10–4.50%, respectively.

## Introduction

Metal-organic frameworks (MOFs) are considered to be a progressively developed hybrid organic-inorganic supramolecular material consisting of organic linkers linking metal ions and metal oxide clusters together through 2D or 3D structures. Due to its characteristics of good stability, adjustable pores, large specific surface area, and polymetallic sites, it has been found that MOFs can be used for sensing some analytes such as H_2_O_2_, glucose, dopamine and heavy metal ions (1, 2). Hu et al. (3) used the abundant active sites of MOFs to develop a highly sensitive and highly selective bifunctional electroactive MOFs as probes to detect the target metal ions by replacing the metal ion sites in the original MOFs. Lu et al. (4) synthesized UiO-67@Ni-MOF composites through the internal expansion growth of shell Ni-MOF on core UiO-67, which showed high selectivity and sensitivity for the detection of glucose in human serum. Wang et al. (5) synthesized two MOF materials by hydrothermal procedure, that can be used to detect trace amounts of Cr_2_O_7_^2–^, Fe^3+^, and Hg^2+^. Up to date, there are no reports about stable and sensitive AuMOF resonance Rayleigh scattering (RRS) nanoprobes for sulfite.

RRS is a simple, rapid and sensitive spectral method, which has been used in the analysis of inorganic and organic substances, such as proteins, nucleic acids, inorganic ions and trace metals (6). Nanoparticles have excellent optical properties, and a series of new RRS methods have been developed based on their surface plasmon RRS effect (7–11). Silicate (SiO_3_^2–^) and phosphate (PO_4_^3–^) can react with ammonium molybdate to form silicomolybdate heteropoly acid (SiMo) and phosphomolybdate heteropoly acid (PMo), PMo decomposed by the addition of citric acid, and SiMo/PMo combined with N/Au co-doped carbon dots (CD_*N/Au*_) show good RRS analytical properties. With the increase of SiO_3_^2–^, SiMo/PMo reacted with CD_*N/Au*_ probe to form large particles, resulting in enhanced RRS intensity at 555 nm. Based on this, Li et al. established a new RRS-ET method to detect SiO_3_^2–^ and PO_4_^3–^ continuously (12). In recent years, a series of new methods of RRS energy transfer (RRS-ET) have been developed to expand the analysis scope, and some substances that cannot be measured by RRS can be realized by means of RRS-ET method. Formaldehyde was condensed with 4-amino-3-homo-5-mercapto-1,2,4-triazolium to produce 6-mercapto-5-triazolium (4,3-b)-S-tetrazolium purplish red compound. The RRS-ET occurs when the compound receptor contacts with the gold nanoparticle donor, leading to the quenching of Rayleigh scattering signal to establish a RRS-ET method for 0.25–64 μmol/L formaldehyde (13). To improve the stability of nanoparticle donor, the liquid crystal, MOFs and covalent organic frameworks (COFs) were studied (13–16), those were not easy to be accumulated by salt comparing to metal nanoparticles. The stable cholesterol benzoate (CB) nanoparticle was used as donor with a strong RRS peak at 395 nm, ammonium ions reacted with acetylacetone and formaldehyde to form a complex 3,5 diacetyl-1,4 dihydrolutidine (DDL) as receptor. Based on the RRS-ET between CB and DDL, a simple RRS-ET platform was established for the determination of trace ammonium (14). The RRS spectra of donor Ce-MOF and absorption spectra of acceptor DDL overlap within 325 –500 nm, and a RRS-ET method for formaldehyde was reported by Chen et al. (15).

With the steady development of society, people’s focus on food has shifted from the initial realization of food and clothing to the pursuit of experience. Preservatives, antioxidants, antibacterial agents, etc. are added in food production to counteract the deterioration of stored food and maintain a good color and luster of the food (17). However, the effect of containing sulfite (SO_3_^2–^) is a double-edged sword. A large number of studies have proved that excessive intake of SO_3_^2–^ will not only induce emergency and various respiratory diseases in some individuals, but also induce cardiovascular diseases and neurological disease, such as abdominal pain, urticaria, asthma, respiratory lung cancer, myocardial ischemia and brain cancer. In food processing, the oxidation of sulfur dioxide and sulfites can effectively inhibit the non-enzymatic browning; with the reducing and bleaching properties of tetravalent S, it can also be used as a preservative to inhibit the growth of mold and bacteria. Therefore, in the production and processing of food, sulfur dioxide and sulfites are often added to make the food fade and free from browning, improve the appearance quality and extend the shelf life. Once these additives are used in excess and without follow-up sulfur dioxide removal technology, it will inevitably lead to excessive sulfur dioxide residues. This will not only damage the quality of the food, but also seriously affect the health of consumers. Because of its potential toxicity, many countries have strictly limited the amount of sulfites in food. The Joint Expert Committee on Food Additives (JECFA) of Food and Agriculture Oganization (FAO)/World Health Organization (WHO) has assessed the risk of sulfur dioxide as a food additive as follows: the Acceptable Daily Intake (ADI) of sulfur dioxide is 0–0.700 mg/kg (as SO_2_) (18). In addition to this, air pollution has also received increasing attention due to its negative impact on human health and environmental quality. Air pollution is mainly caused by various air pollutants emitted by industrial production and transportation, among which SO_2_ is one of the main air pollutants. SO_2_ is a colorless gas under normal conditions, with a pungent odor and poisonous. It is easily soluble in water, dissolves with water and reacts chemically to form sulfurous acid, which is considered to be one of the most dangerous chemicals. In order to identify the potentially polluting environment of SO_2_, a series of materials for identification and detection of SO_2_ were developed. Tchalala et al. (19) developed a fluorinated MOF based on its properties of adsorbing or desorbing molecules, and coated it on a quartz crystal microbalance to achieve the detection of 25.0–500 ppm SO_2_. For the first time, DMello et al. (20) reported that MOF was transformed into a chemiresistive sensing platform for trace detection of SO_2_. In recent years, the methods used to detect sulfite and SO_2_ in food mainly include titrimetric analysis, flow injection analysis, voltammetric analysis, ion chromatography, chemiluminescence, gas chromatography, fluorimetry, high performance liquid chromatography and colorimetric methods (21). For example, Zhang et al. (22) used melamine nanogold as a probe to detect sulfite and hypochlorite by colorimetric analysis. Yuan et al. (23) prepared an imidazo[1,5-α]pyridine-derivated fluorescence sensor for detection of sulfite, with a detection limit of 50 nmol/L. The above methods have advantages, but also have some shortcomings. For example, some methods are simple but low sensitivity; some good sensitivity but the experimental operation is too complex, and the experimental equipment used is more expensive, so it is necessary to develop a selective, highly sensitive, rapid and simple method for detection of sulfite and SO_2_. However, the determination of sulfite by AuMOF RRS-ET method has not been reported so far. In this experiment, it was found that the contact between AuMOF and BF would produce a RRS-ET at the surface of AuMOF, resulting in a reduction of the Rayleigh scattering signal. Accordingly, a new and sensitive RRS-ET method for the determination of SO_3_^2–^ in food was established.

## Apparatus and reagents

### Apparatus

A model of H-800 transmission electron microscope (Hitachi, Japan), with a dot pitch of 0.45 nm, an acceleration voltage of 200 KV, and a tilt angle of ± 25^°^, a model of FEI Talos F200S field emission transmission electron microscope (Thermo Fisher Scientific, Massachusetts, USA), a model of F-7000 fluorescence spectrophotometer (Hitachi, Japan), a model of TU-1901 dual-beam ultraviolet-visible spectrophotometer (Beijing General Analysis General Instrument Co., Ltd., Beijing, China), a model of C-MAG HS7 heating magnetic stirrer (IKA, Germany), a model of constant temperature magnetic stirrer (Beijing Kewei Yongxing Instrument Co., Ltd., Beijing, China), a model of KC-6120 atmospheric integrated sampler (Qingdao Laoshan Electronic Instrument General Factory Co., Ltd., Qingdao, China) were used.

### Reagents

A 0.0126 g of sodium sulfite was dissolved in 10.0 mL water to obtain 0.0100 mol/L sodium sulfite stock solution, and store away from light before being diluted to 1.00 × 10^–3^mol/L or 1.00 × 10^–4^mol/L when used. A 1.00 × 10^–3^mol/L basic fuchsin (BF) solution was prepared as follows, weigh 8.10 mg BF and dissolve in 25.0 mL volumetric flask, then fix the volume and shake well. Dissolve with ultrasound and dilute to 1.00 × 10^–5^mol/L when used. A 100 mmol/L pH 7.50 4-hydroxyethyl piperazine sodium ethanesulfonate/hydrochloric acid buffer solution (HEPES-HCl) was prepared as follows, 0.260 g HEPES was dissolved in water, then 370 μL of 1.00 mol/L HCl was added and the volume was fixed to 10.0 mL. 0.0100 mol/L silver nitrate solution, 30.0% H_2_O_2_ solution, 0.100 mol/L sodium borohydride solution (ready to use), 1.00% chloroauric acid solution (HAuCl_4_, Sinopharm Chemical Reagent Co., Ltd., China), and 1.00% trisodium citrate solution were used. Anhydrous ethanol (C_2_H_5_OH, Guangdong Guanghua Technology Co., Ltd.), 1,2,4-benzenetricarboxylic acid (BTA, Shanghai Macklin Biochemical Co., Ltd.), N,N-dimethylformamide (DMF, Sichuan Xilong Science Co., Ltd.) and triethylamine (TEA, Xilong Science Co., Ltd.) were also used in this experiment. The reagents used were all analytically pure, and the water used for the experiments was secondary distilled water.

### Preparation of Au metal organic frameworks

A 1 mmol BTA and a 1 mmol HAuCl_4_ was separately dissolved in 20 mL DMF/C_2_H_5_OH/H_2_O (1:1:1 v/v) before the two solutions were mixed with stirring. Then 0.100 mL of 7.00 mol/L TEA was added, and the synthesis mixture was placed in a Teflon jar, sealed and irradiated in a microwave oven at 110 °C for 4.00 h. After the reaction, it was cooled to room temperature, and the solid product was obtained by centrifugation. The solid product was washed with DMF/H_2_O to remove unreacted BTA, and dried at 100 °C for 12.0 h. The above synthesis method for preparing AuMOF is the original method, and it has not been reported to use this method to synthesize AuMOF by this method.

### Preparation of gold nanosol (AuNP)

AuNP: 100 mL water was placed in a clean conical flask and boiled, then 7.00 mL of 1.00% aqueous sodium citrate was quickly added, and 1.00 mL of 1.00% HAuCl_4_ was added under stirring. The solution was continued to boil for 10.0 min and removed, stirred and cooled to room temperature under no heating conditions. The volume was fixed to 100 mL and stored in a sterile seal. The particle size of the colloidal gold was about 10.0 nm. The concentration of the gold nanoparticles was 58.0 μg/mL (0.290 mmol/L).

### Preparation of silver nanosol (AgNP)

AgNP: 40.0 mL water was added into a 50.0 mL conical flask, and stirred with a magnetic stirrer while adding 3.50 mL of 10.0 g/L trisodium citrate and 385 μL of 2.40 × 10^–2^ mol/L AgNO_3_. After mixing thoroughly, 4.00 mL of 0.500 mg/mL sodium borohydride was added slowly drop by drop, and the solution changes from light yellow to dark yellow. Keep stirring for 10.0 min before the solution was fixed to 50.0 mL and stored at 4.00 °C. The concentration of the silver nanoparticles was 0.180 mmol/L.

### Experimental procedures

A 100 μL of PBS buffer solution at pH 6.80, 150 μL of 1.00 × 10^–5^ mol/L BF, and a certain volume of 1.00 × 10^–4^ mol/L SO_3_^2–^ was successively added into a 5.00 mL stoppered graduated tube, and shake well. After standing for 2 min, 200 μL of 1.00 μg/mL AuMOF was added, then mixed to 2.00 mL and shake well. The RRS spectra were obtained by simultaneous scanning with a fluorescence spectrophotometer under the conditions of volt = 450 V, excited slit = emission slit = 5.00 nm, emission filter = 1.00% T attenuator, λ_*ex*_–λ_*em*_ = Δλ = 0. The RRS intensity of the solution at 330 nm was measured as I_330 *nm*_, and the intensity of reagent blank without SO_3_^2–^ was recorded as (I_330 *nm*_)_0_. The value of ΔI_330 *nm*_ = I_330 *nm*_-(I_330 *nm*_)_0_ was calculated.

## Results and discussion

### Principle of the method

**FIGURE 1:**
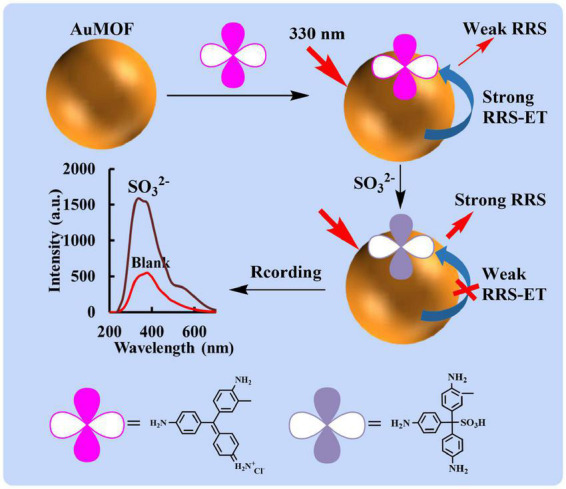
RRS-ET detection of SO_3_^2–^ based on the energy transfer between AuMOF and BF.

In this study, the synthesized AuMOF has a strong RRS signal at 330 nm. When it is used as a nanoprobe, BF is adsorbed on the surface of AuMOF due to intermolecular forces, and AuMOF acts as an energy donor to transfer energy to the acceptor BF. When there is no SO_3_^2–^ in the solution, the addition reaction of BF does not occur, and strong RRS-ET occurs between BF and AuMOF, so the RRS signal at 330 nm was weak. When SO_3_^2–^ was added, which can make BF undergo an addition reaction to form a colorless BF product (SBF) (24). The weak RRS energy transfer between SBF and AuMOF was appeared at 330 nm. Therefore, the RRS signal at 330 nm for SO_3_^2–^-BF-AuMOF analysis system shows an increasing trend with the increase of SO_3_^2–^ concentration (Figure 1). Accordingly, a new method of RRS-ET for the detection of SO_3_^2–^ was established.

### Characterization of Au metal organic frameworks

**FIGURE 2:**
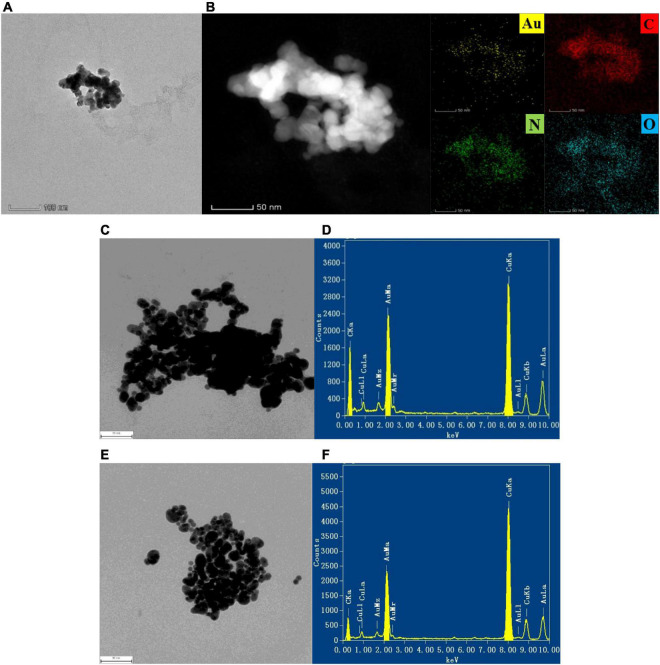
TEM images of AuMOF and the analysis system, the measuring parameters were accelerating voltage = 200 kV, point resolution = 0.250 nm, tilt angle = ± 25.0 degrees. **(A)** TEM image of AuMOF; **(B)** TEM-EDS elemental mapping of AuMOF; **(C)** TEM image of pH 6.80 PBS + 0.750 μmol/L BF + 0.0725 mmol/L AuNP solution; **(D)** EDS of pH 6.80 PBS + 0.750 μmol/L BF + 0.0725 mmol/L AuNP solution; **(E)** TEM image of pH 6.80 PBS + 0.750 μmol/L BF + 2.50 μmol/L SO_3_^2–^ + 0.0725 mmol/L AuNP solution; **(F)** EDS of pH 6.80 PBS + 0.750 μmol/L BF + 2.50 μmol/L SO_3_^2–^ + 0.0725 mmol/L AuNP solution.

The transmission electron microscopy (TEM) of the system and AuMOF were obtained according to the experimental method (Figures 2A,C,E). For AuMOF, it can be seen from Figure 2A that it exhibits a spherical-like morphology. Figure 2B shows that Au, C, N, and O elements are uniformly distributed on the Au-MOF. For the SO_3_^2–^-BF-AuNP system, it can be seen from the TEM results that the aggregation degree of nanogold becomes smaller and smaller as the SO_3_^2–^ concentration increases (Figure 2E). That is, the nanogold slowly changes from the aggregated state to the dispersed state, which is due to the fact that the BF can make the AuNP aggregate under the pH 6.80 PBS buffer condition. As the SO_3_^2–^ concentration increases, more BF was reacted off, thus making the degree of nanogold aggregation slowly become smaller. The energy spectrum (EDS) of the nanogold system was obtained by transmission electron microscopy (voltage: 200 kV) according to the experimental method (Figure 2D), and the energy spectrum of the SO_3_^2–^-BF-AuNP system was shown (Figure 2F). The EDS spectrum shows a major peak (Au), i.e., a bimetallic nanostructure composed of Au atoms. As can be seen from the figure, the element Au exhibits a major peak at 2.12 keV and the appearance of the copper peak is due to the loading of the sample with a copper mesh.

**FIGURE 3:**
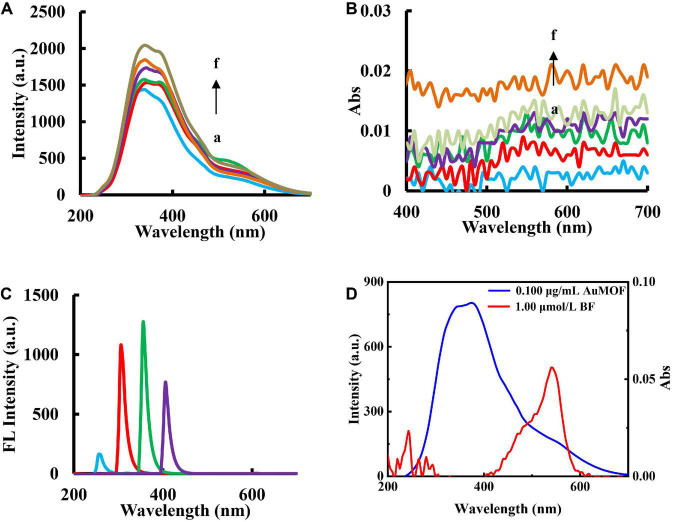
RRS, UV-Vis absorption and fluorescence spectra of AuMOF nanosol. **(A)** a–f curves represent: RRS spectra of (0.100, 0.200, 0.400, 0.800, 20.0, 50.0 μg/mL) AuMOF. **(B)** a–f curves represent: UV-Vis absorption spectra of (0.100, 0.200, 0.400, 0.800, 1.00, 20.0 μg/mL) AuMOF. **(C)** Fluorescence spectra of 0.800 μg/mL AuMOF (excitation wavelengths = 250, 300, 350, 400 nm). **(D)** Relationship between the RRS of AuMOF and the absorption spectrum of BF.

The RRS and UV-Vis absorption spectra of AuMOF nanosol were studied. The results showed that the RRS and Abs intensities linearly increased with the concentration of AuMOF (Figures 3A,B). In addition, the fluorescence spectra of AuMOF was also investigated by taking 0.800 μg/mL AuMOF as an example. The corresponding emission peak was recorded with excitation wavelengths of 250, 300, 350 and 400 nm, respectively, under the voltage of 500 V (Figure 3C). The peak was ascribing to Rayleigh scattering peak of the nanoparticles, so AuMOF has no fluorescence peak. It can be seen from Figure 3D that the absorption spectra of BF overlaps with the RRS spectra of AuMOF in the range of 400–600 nm. This shows that AuMOF can be used as a nanoprobe to achieve RRS-ET between BF and AuMOF.

**FIGURE 4:**
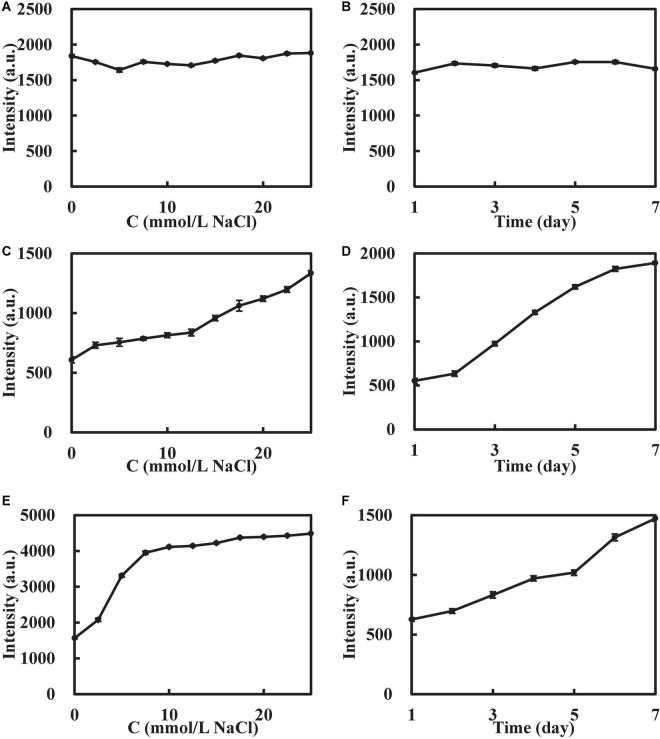
Stability of the nanosol. **(A)** Effect of NaCl on I_330 *nm*_ of AuMOF. **(B)** Effect of time on I_330 *nm*_ of AuMOF. **(C)** Effect of NaCl on I_370 *nm*_ of AuNP. **(D)** Effect of time on I_370 *nm*_ of AuNP. **(E)** Effect of NaCl on I_370 *nm*_ of AgNP. **(F)** Effect of time on I_370 *nm*_ of AgNP. The concentrations of AuMOF, AuNPs, and AgNPs were 20.0 μg/mL, 21.6 μmol/L, and 13.5 μmol/L respectively.

In this experiment, the stability of AuMOF/AuNP/AgNP was tested. The AuMOF was configured as a homogeneous suspension with a concentration of 20.0 μg/mL, placed at room temperature (25°C) for 7 days, and the RRS signal of changes at different times were recorded (Figure 4). The results show that the RRS intensity of 330 nm (I_330 *nm*_) did not fluctuate greatly within 7 days, indicating that the prepared AuMOF had good stability. A 21.6 μmol/L AuNP and 13.5 μmol/L AgNP were placed in the same environment for 7 days, and the signal changes of I_370 *nm*_ at different times were recorded. The results show that the I_370 *nm*_ of AuNP/AgNP increased with time in 7 days, indicating that the prepared AuNP/AgNP continued to aggregate with time. In addition, the electrolyte resistance of AuMOF/AuNP/AgNP were also investigated. A 400 μL of 0.100 g/L AuMOF, 150 μL of 0.290 mmol/L AuNP, and 150 μL of 0.180 mmol/L AgNP solution were added to 3 groups of glass tubes, respectively, with 11 glass tubes in each group. A 0–500 μL of 0.100 mol/L NaCl solution was added to each group to make up to 2.00 mL. The results show that the RRS of AuMOF remained stable when the NaCl concentration was as high as 25.0 mmol/L, while the I_370n*m*_ of AuNP/AgNP increased with the increase of NaCl, indicating that NaCl would induce the aggregation of AuNP/AgNP. Therefore, AuMOF has good electrolyte resistance compared to AuNP/AgNP. So, the AuMOF nanosol was chosen for the analytical experiment.

### Resonance Rayleigh scattering and absorption spectra of analytical system

**FIGURE 5:**
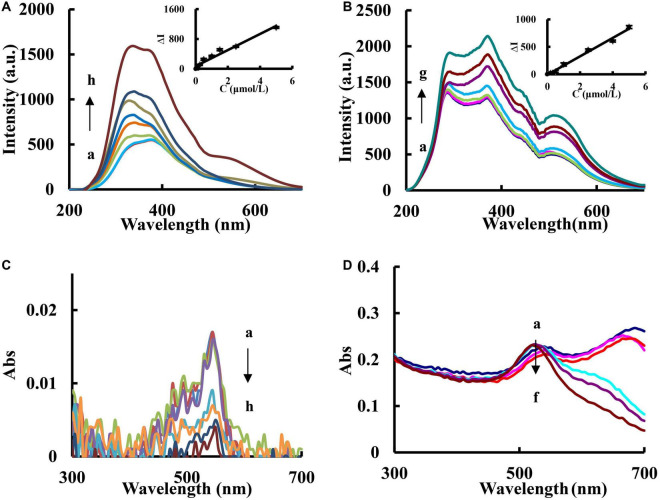
RRS and UV-Vis absorption spectra of SO_3_^2–^-BF-AuMOF/AuNP system. **(A)** RRS spectra of AuMOF system, (a) pH 6.80 PBS + 0.750 μmol/L BF + 0.100 μg/mL AuMOF; (b) a + 0.160 μmol/L SO_3_^2–^; (c) a + 0.250 μmol/L SO_3_^2–^; (d) a + 0.500 μmol/L SO_3_^2–^; (e) a + 1.00 μmol/L SO_3_^2–^; (f) a + 1.50 μmol/L SO_3_^2–^; (g) a + 2.50 μmol/L SO_3_^2–^; (h) a + 3.00 μmol/L SO_3_^2–^. **(B)** RRS spectra of AuNP system, (a) pH 6.80 PBS + 0.750 μmol/L BF + 0.0725 mmol/L AuNP; (b) a + 0.100 μmol/L SO_3_^2–^; (c) a + 0.500 μmol/L SO_3_^2–^; (d) a + 1.00 μmol/L SO_3_^2–^; (e) a + 2.50 μmol/L SO_3_^2–^; (f) a + 4.00 μmol/L SO_3_^2–^; (g) a + 5.00 μmol/L SO_3_^2–^. **(C)** UV-Vis absorption spectra of AuMOF system, (a) pH 6.80 PBS + 0.750 μmol/L BF + 0.100 μg/mL AuMOF; (b) a + 1.00 μmol/L SO_3_^2–^; (c) a + 1.50 μmol/L SO_3_^2–^, (d) a + 2.50 μmol/L SO_3_^2–^; (e) a + 15.0 μmol/L SO_3_^2–^; (f) a + 25.0 μmol/L SO_3_^2–^; (g) a + 35.0 μmol/L SO_3_^2–^; (h) a + 40.0 μmol/L SO_3_^2–^. **(D)** UV-Vis absorption spectra of AuNP system, (a) pH 6.80 PBS + 0.750 μmol/L BF + 0.0725 mmol/L AuNP; (b) a + 0.250 μmol/L SO_3_^2–^; (c) a + 1.00 μmol/L SO_3_^2–^, (d) a + 4.00 μmol/L SO_3_^2–^; (e) a + 6.00 μmol/L SO_3_^2–^; (f) a + 10.0 μmol/L SO_3_^2–^.

In the analytical system, there is a strong RRS peaks at 330 nm for AuMOF and 370 nm for AuNP (Figures 5A,B). The SO_3_^2–^ reacted with BF to form colorless addition reaction product SBF. Due to the RRS energy transfer between AuMOF/AuNP and BF, as the concentration of SO_3_^2–^ increases, the BF in the system decreases, and the energy transferred from AuMOF/AuNP decreases. However, the RRS-ET between AuMOF/AuNP and BF was weak. The peak intensity at 330 nm increased linearly with the increase of SO_3_^2–^ concentration. So, the peak at 330 nm for AuMOF system and 370 nm for AuNP system were chosen to determine SO_3_^2–^ in this experiment. For the AuMOF system, it is a decreasing trend for the absorption peak at 540 nm produced by BF (Figure 5C) (25). For the AuNP system, BF produced an addition reaction with SO_3_^2–^, and there was an absorption peak at 540 nm (Figure 5D). However, due to the low concentration of BF and high concentration of AuNP in this experiment, the characteristic absorption peak of AuNP was more obvious and that of BF absorption change was not obvious. So, the absorption cannot be used to determine SO_3_^2–^.

### Selection of analysis conditions

The analysis conditions were studied by the univariate method, and the optimal analysis conditions were selected. In the experiment, a blank control group without SO_3_^2–^ and an experimental group with a certain amount of SO_3_^2–^ were set up, the RRS signal intensities I_0_ and I were measured, respectively, and the difference ΔI was calculated. By changing the conditions to be optimized and comparing ΔI, the optimal analysis conditions can be screened. As can be seen from Supplementary Figure 1A, the pH of the PBS buffer solution was chosen to be 6.80. The effect of the PBS buffer solution concentration on the system ΔI was investigated. Because phosphate in the system also plays the role of ionic strength agent, when the concentration of PBS in the system is too small, the reaction does not proceed, that is, there will be a situation that the blank value larger than the reaction system. The ΔI was maximum when the PBS concentration was 10.0 mmoL/L, so the PBS concentration was chosen to be 10.0 mmoL/L. The effect of BF concentration on the ΔI of the system was studied. As shown in Supplementary Figure 1B, ΔI was maximum when the concentration of BF solution was 0.750 μmol/L, so 0.750 μmol/L BF solution was selected. The effect of AuMOF concentration on the system ΔI was also investigated. When the concentration of AuMOF solution was 0.100 μg/mL, ΔI was the largest, so AuMOF solution of 0.100 μg/mL was selected (Supplementary Figure 1C). The effect of AuNP concentration on the ΔI of the system was discussed too. As shown in Supplementary Figure 1D, ΔI was maximum when the AuNP solution concentration was 0.0725 mmol/L, so the AuNP solution concentration was chosen to be 0.0725 mmol/L. The effect of the reaction time of SO_3_^2–^ with BF was investigated. Two min later, the reaction was basically complete, so AuMOF/AuNP was added after the addition of SO_3_^2–^ and left for 2 min.

### Working curves

The working curves of the three nanoprobes were plotted according to the experimental method (Table 1). The detection limit of the method was determined based on the 3s/m criterion (where s was the standard deviation of the blank or standard deviation of the intercept and m was the slope of the calibration plot), and the number of blank samples was greater than 10. For the RRS system of SO_3_^2–^-BF-AuMOF, the slope of working curve was largest and the detection limit (DL) was lowest, with a linear range (LR) of 0.160–5.00 μmol/L. What’s more, the AuMOF nanoprobe stability was better than the AuNP and AgNP. Thus, it was chosen for the determination of sulfite. Compared with the reported methods for the determination of sulfite (26–31), the method of RRS-ET in this work has the advantages of simple operation, high sensitivity and good selectivity (Supplementary Table 1).

**TABLE 1:** Comparison of analytical properties of the three nanoprobes.

Nanoprobes	Method	Regression equation	LR (μmol/L)	Coefficient	DL (μmol/L)
AuMOF	RRS	ΔI = 215C + 72.7	0.160–5.00	0.959	0.0800
AuNP	RRS	ΔI = 168C- 7.90	0.200–5.00	0.995	0.100
AgNP	RRS	ΔI = 60.7C + 61.0	0.200–20.0	0.995	0.100

### Effect of coexisting substances

The interference of commonly coexisting substances was investigated according to the experimental procedure. The results showed that when the relative errors were within ± 10%, 100 times of Al^3+^, Zn^2+^, Ca^2+^, Co^2+^, Bi^+^, Cu^2+^, Ba^2+^, Mg^2+^, K^+^, Ni^2+^, NO^2–^, SO_4_^2–^, TeO_4_^2–^, SeO_3_^2–^, benzoic acid, phenylhydroxyethylic acid, 25.0 times of Mn^2+^ and 2.00 times of Na_2_S, had little effect on the measurement results of 2.50 μmol/L sulfite (Supplementary Table 2). It indicates that the system has a good selectivity for the determination of SO_3_^2–^, so this system was chosen to analyze the samples in this paper.

### Analytical application

The production of sulfur dioxide in food mainly comes from sulfite used in food processing. Sulfite is a class of bleaching agents that work to produce sulfur dioxide to destroy or inhibit the coloring factors in food to make it fade or save it from browning. Sulfite can be used in salted vegetables, sauerkraut, edible starch, starchy sugar, chocolate, semi-solid compound seasoning, fruit and vegetable juices, wine, fruit wine, beer and malt beverages. According to the China national standard GB2760-2011, the maximum sulfur dioxide residue in fruit and vegetable juice is 0.0500 g/kg, in beer is 0.0100 g/kg, and in fruit wine is 0.250 g/kg. The contents of sulfite (SO_3_^2–^) in different kinds of beer and fruit and vegetable juices samples that bought from the market were analyzed according to the procedures. The results show that the sulfite residues in commercially available samples of draft beer and fruit and vegetable juices exceeded the standard, while the sulfite residues in pineapple beer and apple cider vinegar were within the standard (Table 2). The recoveries of the four samples were between 97.1 and 106%, with relative standard deviations (RSD) of 2.10–4.80%.

**TABLE 2:** Results for determination of SO_3_^2–^ in food samples.

Sample	Single value (μmol/L)	Average (μmol/L)	Content (g/kg)	Added (μmol/L)	Found (μmol/L)	Recovery (%)	RSD (%)
Pineapple beer	2.54 2.53 2.66 2.54 2.44	2.55	5.10 × 10^–3^	0.500	2.98	97.7	3.80
				1.00	3.69	104	4.10
Draft beer	3.28 3.42 3.56 3.31 3.58	3.43	0.0180	0.500	4.08	104	2.80
				1.00	4.30	97.1	2.40
Fruit and vegetable juice	1.81 1.80 1.88 1.78 1.73	1.80	0.230	0.500	2.43	106	2.10
				1.00	2.75	98.1	3.80
Apple cider vinegar	0.98 1.07 0.97 1.05 1.00	1.02	0.0130	0.500	1.54	103	4.80
				1.0	2.11	106	3.50

The sulfur dioxide (SO_2_) contents in the air samples of inside the laboratory (Samples 1, 2, 3) and outside the laboratory (Sample 4) were examined according to the experimental method, respectively. Sampling procedure was as follows: 5.00 mL of 5.00 mmol/L absorbing solution was added into the U-shaped absorbing tube before it was connected to the atmospheric sampler, and sampled at a flow rate of 0.500 L/min for 20 min. Then the sampled solution was transferred to a 5.00 mL test tube, and a blank control was used for SO_2_ concentration detection. According to China National Standard GB/T17097-1997 for sulfur dioxide in indoor air, the daily average maximum permissible concentration of sulfur dioxide in indoor air should not exceed 0.150 mg/m^3^. As shown in Supplementary Table 3, the sulfur dioxide content in the four samples collected did not exceed the standard. The results of the spiked recovery experiments for four samples are shown in Supplementary Table 3, with recoveries between 92.9 and 106% and RSDs between 2.10 and 4.50%.

## Conclusion

A new, stable and strong RRS AuMOF nanoprobe was fabricated and characterized by electron microscopy and molecular spectral techniques. Based on the principle of addition reaction between sulfite and BF to form colorless SBF, strong RRS-ET between AuMOF donor and BF receptor, and weak RRS-ET between AuMOF and SBF, a new RRS-ET method was established for detecting SO_3_^2–^. Compared with the reported methods of SO_3_^2–^ (17, 30, 32–34), this method is simple and fast since large, expensive instruments and complex operations are not required. What’s more, it is sensitive with the linear range of 0.160–5.00 μmol/L SO_3_^2–^ and DL of 0.0800 μmol/L. It can meet the requirement of sulfite determination in food and other samples with satisfactory results. Finally, it is selective and less affected by other coexisting ions according to the interference experiment of 18 substances. This method was used for the determination of SO_3_^2–^ in real food samples and SO_2_ in air, and the results are accurate. So, the method has the advantages of simplicity, rapidness, sensitivity and selectivity. Although this assay is sensitive, there is a need to improve linear range and feasibility for broad applicability. In the future, more kinds of nanomaterials (metal nanoparticles, COF, MOF, atomic clusters, etc.) will be developed to study RRS-ET to achieve the detection of target substance, to develop high-sensitivity methods and multimodal methods.

## Data availability statement

The original contributions presented in this study are included in the article/Supplementary material, further inquiries can be directed to the corresponding author.

## Author contributions

XL: formal analysis, data curation, and writing—original draft preparation. YL and SZ: data curation and writing—original draft preparation. MW: writing—original draft preparation. GW and ZJ: conceptualization, formal analysis, supervision, project administration, funding acquisition, and writing—review and editing. All authors contributed to the article and approved the submitted version.

## References

[1] Samuel MS Bhattacharya J Parthiban C Viswanathan G Singh NDP. Ultrasound-assisted synthesis of metal organic framework for the photocatalytic reduction of 4-nitrophenol under direct sunlight. *Ultrason Sonochem.* (2018). 49:215–21. 10.1016/j.ultsonch.2018.08.004 30150024

[2] Samuel MS Subramaniyan V Bhattacharya J Parthiban C Chand S Singh NDP. A GO-CS@MOF [Zn (BDC)(DMF)] material for the adsorption of chromium (VI) ions from aqueous solution. *Compos B Eng.* (2018) 152:116–25. 10.1016/j.compositesb.2018.06.034

[3] Hu R Zhang X Chi KN Yang T Yang YH. Bifunctional MOFs-based ratiometric electrochemical sensor for multiplex heavy metal ions. *ACS Appl Mater Interfaces.* (2020) 12:30770–8. 10.1021/acsami.0c06291 32497422

[4] Lu M Deng Y Li Y Li T Xu J Chen SW Core-shell MOF@ MOF composites for sensitive nonenzymatic glucose sensing in human serum. *Anal Chim Acta.* (2020) 1110:35–43. 10.1016/j.aca.2020.02.023 32278398

[5] Wang ZJ Ge FY Sun GH Zheng HG. Two MOFs as dual-responsive photoluminescence sensors for metal and inorganic ion detection. *Dalton Trans.* (2018) 47:8257–63. 10.1039/c8dt01363b 29888352

[6] Wang J Liu S Shen W. Absorption and resonance Rayleigh scattering spectra of Ag (I) and erythrosin system and their analytical application in food safety. *Front Nutr.* (2022) 9:900215. 10.3389/fnut.2022.900215 35614984 PMC9125220

[7] Garg D Rekhi H Kaur H Singh K Malik AK. A novel method for the synthesis of MOF-199 for sensing and photocatalytic applications. *J Fluoresc.* (2022) 32:1171–88. 10.1007/s10895-022-02902-9 35347530

[8] Li J Wang J Zhang X Chang H Wei W. Highly selective detection of epidermal growth factor receptor by multifunctional gold-nanoparticle-based resonance Rayleigh scattering method. *Sens Actuators B.* (2018) 273:1300–6. 10.1016/j.snb.2018.07.046

[9] Yao DM Wen GQ Gong LB Li CN Liang AH Jiang ZL. A highly sensitive SERS and RRS coupled di-mode method for CO detection using nanogolds as catalysts and bifunctional probes. *Nanomaterials.* (2020) 10:450. 10.3390/nano10030450 32131528 PMC7153473

[10] Wang HL Zhang ZH Chen CQ Liang AH Jiang ZL. Fullerene carbon dot catalytic amplification-aptamer assay platform for ultratrace As^+3^ utilizing SERS/RRS/Abs trifunctional Au nanoprobes. *J Hazard Mater.* (2021) 403:123633. 10.1016/j.jhazmat.2020.123633 32827860

[11] Li D Li CN Wang HL Li J Zhao YX Jiang X Single-atom Fe catalytic amplification-gold nanosol SERS/RRS aptamer as platform for the quantification of trace pollutants. *Microchim Acta.* (2021) 188:175. 10.1007/s00604-021-04828-8 33893886

[12] Li J Li CG Zhang ZH Wang XY Liang AH Wen GQ A novel N/Au co-doped carbon dot probe for continuous detection of silicate and phosphate by resonance Rayleigh scattering. *Analyst.* (2019) 144:5090–7. 10.1039/c9an01072f 31360936

[13] Wen GQ Liang XJ Liang AH Jiang ZL. Gold nanorod resonance Rayleigh scattering-energy transfer spectral determination of trace formaldehyde with 4-amino-3-hydrazino-5-mercap-1, 2, 4-triazole. *Plasmonics.* (2015) 10:1081–8. 10.1007/s11468-015-9893-6

[14] Lv XW Liao LP Chen SX Xiao Y Jiang ZL Wen GQ. A cholesterol benzoate RRS probe for the determination of trace ammonium ions. *Spectrochim Acta Part A.* (2022) 272:120945. 10.1016/j.saa.2022.120945 35151166

[15] Chen SX Wu ML Lv XW Xiao Y Jiang ZL Wen GQ. A novel resonance Rayleigh scattering assay for trace formaldehyde detection based on Ce-MOF probe and acetylacetone reaction. *Microchem J.* (2022) 179:107501. 10.1016/j.microc.2022.107501

[16] Shi JL Wang HL Ma XT Liang AH Jiang ZL. A facile COF loaded-molybdate resonance Rayleigh scattering and fluorescence dimode probe for determination of trace PO_4_^3–^. *Spectrochim Acta Part A.* (2022) 280:121500. 10.1016/j.saa.2022.121500 35738110

[17] Venkatachalam K Asaithambi G Rajasekaran D Periasamy V. A novel ratiometric fluorescent probe for “naked-eye” detection of sulfite ion: applications in detection of biological SO_3_^2–^ ions in food and live cells. *Spectrochim Acta Part A.* (2020) 228:117788. 10.1016/j.saa.2019.117788 31757702

[18] Koch M Köppen R Siegel D Witt A Nehls I. Determination of total sulfite in wine by ion chromatography after in-sample oxidation. *J Agric Food Chem.* (2010) 58:9463–7. 10.1021/jf102086x 20690603

[19] Tchalala MR Bhatt PM Chappanda KN Tavares SR Adil K Belmabkhout Y Fluorinated MOF platform for selective removal and sensing of SO_2_ from flue gas and air. *Nat Commun.* (2019) 10:1328. 10.1038/s41467-019-09157-2 30902992 PMC6430820

[20] DMello ME Sundaram NG Singh A Singhc AK Kalidindi SB. An amine functionalized zirconium metal–organic framework as an effective chemiresistive sensor for acidic gases. *Chem Commun.* (2019) 55:349–52. 10.1039/c8cc06875e 30534782

[21] Pundir CS Rawal R. Determination of sulfite with emphasis on biosensing methods: a review. *Anal Bioanal Chem.* (2013) 405:3049–62. 10.1007/s00216-013-6753-0 23392406

[22] Zhang J Xu X Yang X. Role of tris on the colorimetric recognition of anions with melamine-modified gold nanoparticle probe and the visual detection of sulfite and hypochlorite. *Analyst.* (2012) 137:3437–40. 10.1039/c2an35609k 22673941

[23] Yuan Q Chen LL Zhu XH Yuan ZH Duan YT Yang YS An imidazo[1,5-α]pyridine-derivated fluorescence sensor for rapid and selective detection of sulfite. *Talanta.* (2020) 217:121087. 10.1016/j.talanta.2020.121087 32498830

[24] Zhang WD. Kinetic spectrophotometric determination of trace sulfite based on its addition reaction with rosaniline. *Chin J Anal Chem.* (1995) 23:1405–8.

[25] Shagufta NAZ. Aqueous solution of basic fuchsin as food irradiation dosimeter. *Nucl Sci Tech.* (2007) 18:141–4. (07)60035-9 10.1016/S1001-8042

[26] Liu LH Li WB Gao F Huo TR. Adduct of magnesium tetraphenylporphyrin with aniline for colorimetric detection of SO_2_. *Chin Chem Lett.* (2012) 23:208–12. 10.1016/j.cclet.2011.10.018

[27] Yin LQ Yuan DX Zhang M. Determination of sulfite in water samples by flow injection analysis with fluorescence detection. *Chin Chem Lett.* (2010) 21:1457–61. 10.1016/j.cclet.2010.06.029

[28] Wang C Feng S Wu L Yan S Zhong C Guo P A new fluorescent turn-on probe for highly sensitive and selective detection of sulfite and bisulfite. *Sens Actuators B.* (2014) 190:792–9. 10.1016/j.snb.2013.09.045

[29] Yang X Cui Y Li Y Zheng L Xie L Ning R A new diketopyrrolopyrrole-based probe for sensitive and selective detection of sulfite in aqueous solution. *Spectrochim Acta Part A.* (2015) 137:1055–60. 10.1016/j.saa.2014.08.144 25291502

[30] Ju WJ Fu LM Yang RJ Lee CL. Distillation and detection of SO_2_ using a microfluidic chip. *Lab Chip.* (2012) 12:622–6. 10.1039/c1lc20954j 22159042

[31] Zhao Y Wang X Dai D Dong Z Huang Y. Partial discharge early-warning through ultraviolet spectroscopic detection of SO_2_. *Meas Sci Technol.* (2014) 25:035002. 10.1088/0957-0233/25/3/035002

[32] Wang W Li N Liu JT Miao JY Zhao BX Lin ZM. A new FRET-based ratiometric fluorescent probe for the detection of SO_2_ derivatives in mitochondria of living cells. *Dyes Pigm.* (2020) 181:108639. 10.1016/j.dyepig.2020.108639

[33] Urupina D Gaudion V Romanias MN Verriele M Thevenet F. Method development and validation for the determination of sulfites and sulfates on the surface of mineral atmospheric samples using reverse-phase liquid chromatography. *Talanta.* (2020) 219:121318. 10.1016/j.talanta.2020.121318 32887058

[34] Kong D Zhu W Li M. A facile and sensitive SERS-based platform for sulfite residues/SO_2_ detection in food. *Microchem J.* (2021) 165:106174. 10.1016/j.microc.2021.106174

