# Supplementary material for: A stable and sensitive Au metal organic frameworks resonance Rayleigh scattering nanoprobe for detection of SO32– in food based on fuchsin addition reaction
Source: Front Nutr. 2022 Nov 10;9:1019429. doi: 10.3389/fnut.2022.1019429 (PMC9686329; doi:10.3389/fnut.2022.1019429)
Supplement: Supplementary file 1 [file Data_Sheet_1.pdf]

A stable and sensitive Au metal organic frameworks resonance Rayleigh scattering nanoprobe for detection of SO_3^{2-} in food based on fuchsin addition reaction

Xiaowen Lv[1,2], Yue Liu[1,2], Shuangshuang Zhou[1,2], Menglei Wu[1,2], Zhiliang Jiang[1,2], Guiqing Wen[1,2*]

[1]Key Laboratory of Ecology of Rare and Endangered Species and Environmental Protection (Guangxi Normal University), Ministry of Education, Guilin 541004, China; [2]Guangxi Key Laboratory of Environmental Pollution Control Theory and Technology for Science and Education Combined with Science and Technology Innovation Base, Guilin 541004, China.

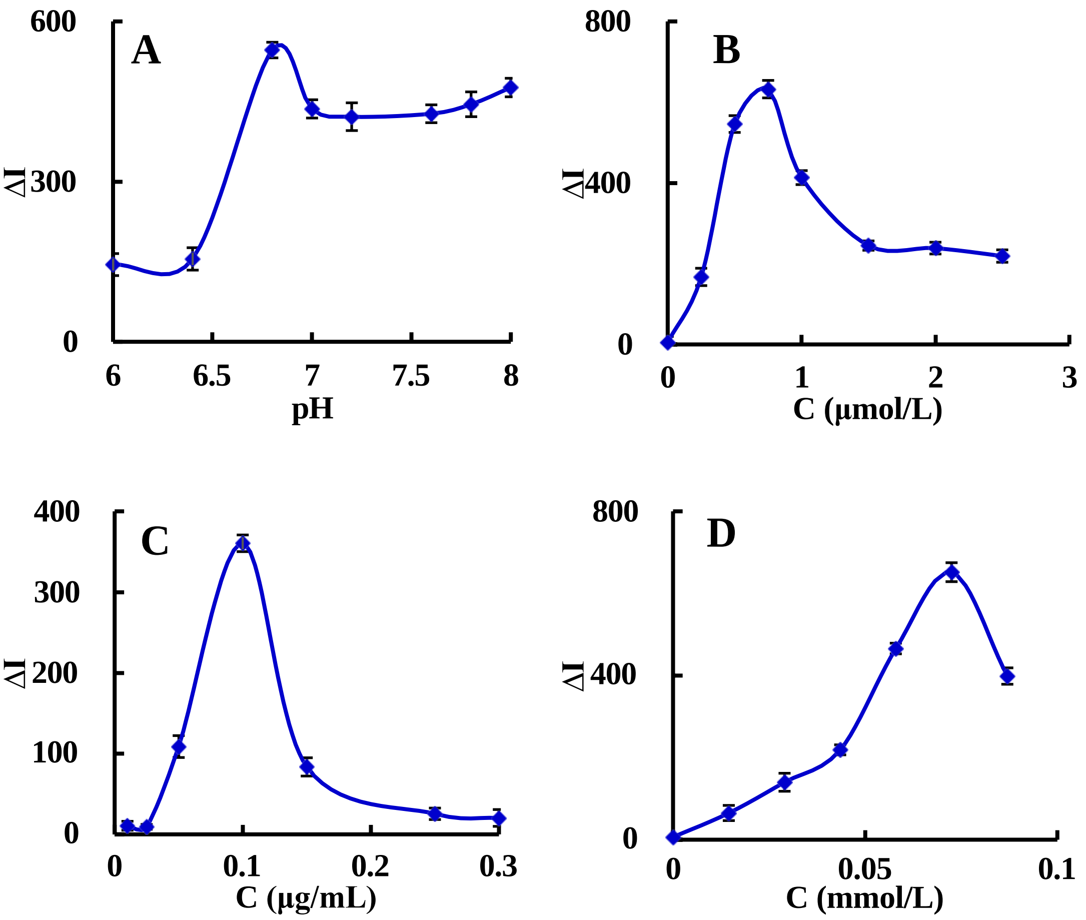

FIGURE S1. Analysis condition selection.

A: The effect of pH of PBS buffer solution, 20.0 mmoL/L PBS + 1.00 μmol/L BF + 25.0 μmol/L SO_3^{2-} + 0.100 μg/mL AuMOF; **B:** The effect of BF concentration, pH 6.80 PBS + BF + 25.0 μmol/L SO_3^{2-} + 0.100 μg/mL AuMOF; **C:** The effect of AuMOF concentration, pH 6.80 PBS + 0.750 μmol/L BF + 25.0 μmol/L SO_3^{2-} + AuMOF; **D:** The effect of AuNP concentration, pH 6.80 PBS + 0.750 μmol/L BF + 25.0 μmol/L SO_3^{2-} + AuNP.

TABLE S1. Comparison of the reported methods for sulfites

Method	Principle	LR	DL	Note	Ref.
Colorimetric method	SO_2 competes with magnesium porphyrin (MgTPP) for aniline, which eventually leads to the color change of MgTPP.	——	—	Complex method and low sensitivity	(26)
	Flow analysis and fluorometric determination of SO_3^{2-} based on the catechol (OPA)-SO_3^{2-} NH_3 reaction.	——	18.0 μmol/L	With good accuracy and anti-	(27)
Fluorescent probes	4-Acylhydrazine-1,8-naphthalimide with sulfite and bisulfite leads to fluorescence enhancement.	——	0.560 μmol/L	interference, the	(28)
	Photometric determination of sulfites based on pyrroloxypyrrolidone doped α,β-unsaturated ketone probes.	——	0.100 μmol/L	Good selectivity and low sensitivity	(29)
Microfluidic chips	A poly(methyl methacrylate) (PMMA) aluminum-based microdistillation chip for SO_2 detection was designed for colorimetric determination of SO_2 concentration.	100-500 ppm	—	Method operation is more complicated	(30)
Photometric method	Based on the decomposition mechanism of sulfur hexafluoride (SF_6), the standard gas of its main decomposition products is detected qualitatively and quantitatively by fiber optic UV spectroscopy. The decomposition experiments are aimed at showing the feasibility of detecting early warnings of SO_2 by UV spectroscopy.	——	—	Complex operation process. Low sensitivity	(31)

RRS	SO_3^{2-} taken place an addition reaction with BF, and it was detected by AuMOF RRS probes.	0.160-5.00 μmol/L	0.0800 μmol/L	Simple method, good selectivity and high sensitivity	This work

TABLE S2. Effect of coexisting substances

Coexistent substance	Tolerance (Times)	Relative error (%)	Coexistent substance	Tolerance (Times)	Relative error (%)
Mn^{2+}	25	9.40	Ni^{2+}	100	-1.90
Al^{3+}	100	1.80	NO^{2-}	100	-4.30
Zn^{2+}	100	7.20	SO_4^{2-}	100	-9.80
Ca^{2+}	100	-0.200	TeO_4^{2-}	100	3.00
Co^{2+}	100	-0.900	SeO_3^{2-}	100	3.80
Bi^{+}	100	-2.00	Benzoic acid	100	3.20
Cu^{2+}	100	-8.50	Phenoxyethanolic acid	100	3.50
Ba^{2+}	100	-8.30	Na_2S	2	7.10
Mg^{2+}	100	-3.30			
K^{+}	100	-0.200			

TABLE S3. Determination results for SO_3^{2-} in air

Samples	SO_2 Single value (μmol/L)	SO_2 Average (μmol/L)	SO_2 Content (mg/m^3)	Add SO_3^{2-} (μmol/L)	SO_3^{2-} (μmol/L)	Recovery (%)	RSD (%)
Sample 1	2.48 2.57 2.50 2.31 2.38	2.45	0.0780	0.500	2.89	98.0	3.60
				1.00	3.60	105	2.20
Sample 2	0.95 0.94 0.91 0.86 0.89	0.910	0.0290	0.500	1.48	105	2.50
				1.00	1.78	92.9	2.10
Sample 3	2.51 2.26 2.41 2.28 2.34	2.36	0.0760	0.500	3.02	106	4.20
				1.00	3.31	98.4	2.60
Sample 4	1.89 2.04 1.87 1.97 1.82	1.92	0.0620	0.500	2.32	96.2	2.30
				1.00	3.00	103	4.50
